# Benefits and Pitfalls of Uraemic Toxin Measurement in Peritoneal Dialysis

**DOI:** 10.3390/jcm14041395

**Published:** 2025-02-19

**Authors:** Aruni Malaweera, Louis Huang, Lawrence McMahon

**Affiliations:** Department of Renal Medicine, Eastern Health, 5, Arnold Street, Box Hill, Melbourne, VIC 3128, Australia; louis.huang@monash.edu (L.H.); lawrence.mcmahon@monash.edu (L.M.)

**Keywords:** uraemic, toxin, urea, creatinine, protein-bound, fiber, adequacy

## Abstract

Chronic kidney disease is a global health burden with a rising incidence and prevalence in developed and developing nations. Once established, it results in a progressive accumulation of a myriad of uraemic toxins. Peritoneal dialysis (PD) uses the body’s peritoneal membrane to remove these toxins across a semipermeable membrane to restore and maintain homeostasis. Traditionally, dialysis adequacy has been measured through clearance of urea and creatinine. However, numerous studies have shown marginal links comparing the clearance of urea and creatinine with clinical outcomes reflected in the recent changes to the ISPD guidelines on dialysis adequacy. Instead, attention has focused on protein-bound uraemic toxins (PBTs). Produced by gut bacteria, these molecules are highly protein-bound and poorly removed by either dialysis or absorptive agents. Elevated concentrations of molecules such as p-cresyl sulfate and indoxyl sulfate have been associated with abnormal cellular function and poor patient outcomes. However, widespread use of these measures to determine dialysis adequacy has been limited by the need for specialized techniques required for measurement. Altering the gut microbiome to reduce generation of PBTs through increased dietary fiber might be an alternate approach to better patient outcomes, with some initial positive reports. This report explores advantages and limitations of measuring uraemic toxins in PD, now and in the foreseeable future.

## 1. Introduction

Kidney disease is a global health burden with up to 7 million people needing renal replacement therapy worldwide [1]. Kidney failure leads to uraemia, an acute or chronic condition signifying an accumulation of uraemic toxins usually removed by the kidneys. Haemodialysis (HD) and peritoneal dialysis (PD) are methods of renal replacement therapy carried out to remove uraemic toxins to maintain homeostasis. Despite dialysis, patients with kidney disease have increased morbidity and mortality, and dialysis patients demonstrate a 500–1000-fold risk of cardiovascular mortality compared with the general population [1,2,3,4,5]. However, uraemia appears more closely related to the high mortality than classic risk factors such as hypertension and hypercholesterolaemia [6]. Likely in relation to this, HD and PD have traditionally assessed the adequacy of their replacement therapy through clearance of urea and creatinine, both of which are small water-soluble molecules easily removed by dialysis.

However, the International Society for Peritoneal Dialysis (ISPD) guidelines in 2020 recommended focusing on the patient’s sense of well-being rather than targeting numerical targets, indicating a shift from the reliance of traditional toxins [7]. Such an approach was driven by evidence that traditional toxins lack robust links to patient outcomes. Moreover, recent studies have suggested that other uraemic solutes such as protein-bound uraemic toxins (PBTs) might better contribute to understanding poorer patient outcomes and abnormal cellular function in chronic kidney disease (CKD).

## 2. Uraemic Toxin Classification

The European Uremic Toxin Work Group (EUTox) identified 90 uraemic toxins by classifying them according to their physiochemical properties; free water-soluble low-molecular-weight solutes (<500 Da), protein-bound solutes, and middle molecules (≥500 Da) [8,9]. Before a uraemic toxin can be accepted as that definition, it needs to comply with a number of conditions: the compound should be chemically identified and able to be measured in biospecimens; the body and plasma quantification of these compounds should be higher in uraemic than non-uraemic patients; high concentrations of these compounds should be associated with symptoms and therefore symptoms decrease when their concentrations have reduced, and finally their biological activity should be proven in vivo, ex vivo, and in vitro studies.

## 3. Urea and Creatinine as Uraemic Toxins

The Euro-tox group defined urea and creatinine as small water-soluble uraemic toxins and therefore their removal has been used in measuring effectiveness of dialysis. However, whether the retention of these solutes leads to direct toxicity and negative patient symptomatology as per the uraemic toxin definition remains inconclusive.

Creatinine, a 113-dalton (Da) molecule, is the product of muscle breakdown where accumulation has been shown to block chloride channels affecting the contractility of myocardial cells. However, this has not been reproduced nor shown to lead to direct toxicity, leading to poorer patient outcomes [10].

Urea is a small 60 Da water-soluble solute that is present in uraemic serum at the highest concentration compared with other solutes. Despite extensive research into its toxicity, urea’s toxic effects have only been demonstrated in a handful of studies where it is said to cause endothelial dysfunction and decrease affinity of oxygen to haemoglobin [11,12,13,14]. However, its negative biological effects on key bodily functions, clinical outcomes, and/or patient symptoms have been debated for decades and never consistently demonstrated in studies [15].

## 4. Use of Urea and/or Creatinine in Dialysis Adequacy

The definition of urea and creatinine as “uraemic toxins”, along with their physiochemical properties, makes it easy and cost-effective to measure (as both creatinine and urea diffuses easily between body compartments and dialysate) and enables them to be the standard dialysis adequacy measure [16]. In peritoneal dialysis, dialysis adequacy using urea and creatinine has universally been assessed with either Kt/V_urea_ or creatinine clearance (CCr). The total weekly Kt/V_urea_ and total creatinine clearance (CCr) are calculated from the combination of weekly peritoneal clearance and renal clearance (using the residual kidney function or RKF). The methods of their measurement are shown in Table 1 [16,17,18,19].

These measures were examined in the CANUSA study, a large multicenter prospective cohort trial of 680 PD patients in the United States and Canada [18]. The author found that a reduction in weekly Kt/V_urea_ by 0.1 or CCr by 5 L/week/1.73 m^2^ led to an increase in relative risk of death by 5 and 7%, respectively. They also found that a reduction in CCr but not Kt/V was associated with increased technique failure and hospitalisation. This led to the guidelines recommending that PD patients should have a minimum weekly Kt/V_urea_ of 1.7 and a CCr of 45 L/week/1.73 m^2^ [2,19,20].

## 5. Limitations in Using Urea and Creatinine in Dialysis Adequacy

Studies in the last two decades have revealed multiple limitations in using urea and creatinine to determine dialysis adequacy. Multiple studies have demonstrated that RKF is a stronger determinant of mortality than peritoneal clearance of urea and/or creatinine [17,20,21,22,23,24,25]. Such studies prompted a re-evaluation of the CANUSA study and found a reduction in relative risk of death by 12% and 36% with every 5 L/week/1.73 m^2^ increase in renal CCr and every 250 mL increase in urine volume, respectively [26]. Such effects were not identified with peritoneal clearance. Since then, larger studies, namely NECOSAD and ADEMEX, have also found no survival advantage with higher CCr or Kt/V_urea_ targets in PD patients and also demonstrated that the rate of decline of RKF has a strong correlation with increased mortality [27,28,29,30,31,32,33]. Additionally, Kt/V_urea_ values did not correlate with uraemic symptoms, technique failure, hospital days, peritonitis rate, or quality of life measures [34,35,36]. While earlier studies on anuric patients have found that Kt/V < 1.85 was associated with higher mortality, more hospitalizations, and technique failure [37,38,39,40,41], these findings were incongruous with later studies that demonstrated no improvement in mortality with enhanced small solute clearance in anuric PD patients [33,42].

In addition, given that urea is the end product of protein catabolism and creatinine is a byproduct of muscle breakdown, adequacy measures based on these molecules might not reflect the patients’ state of renal failure and its improvement with dialysis, but rather their nutritional state and/or muscle mass [43,44]. For example, should a patient stop eating and lose muscle mass, serum urea and creatinine might not rise, nor their dialysis clearance change. Nonetheless, their toxin accumulation increases, and their clinical outcome inevitably worsens. Similarly, differences in diet and/or body mass index may also explain comparative survival outcomes in Asian populations compared to Caucasians despite lower Kt/V results [45].

Finally, increasing the peritoneal dialysis prescription to meet small solute dialysis adequacy targets is associated with increased glucose exposure with glucose-based PD fluids leading to increased metabolic risk, peritoneal membrane failure, vascular dysfunction, and inflammation [46].

Therefore, if urea and/or creatinine removal by dialysis is not consistently correlated with patient outcomes, it is possible that uraemia and its negative effects are related to an accumulation of large range of compounds, rather than a single solute such as urea or creatinine, thus creating a “residual syndrome”. Thus arose the concept of “additive toxicity”, where other uraemic solutes may play a role in uraemic toxicity and its consequences [47]. These include middle molecules such as beta-2-microglobulin (B2M), parathyroid horomone (PTH) and fibroblast growth factor (FGF23), and protein-bound uraemic toxins (PBTs). However, it should also be remembered that rates of urea and creatinine clearance do not necessarily correlate with clearance of other uremic toxins [48].

## 6. Middle Molecules

Defined by their size range of 300–12,000 Da, middle molecules have been associated with uraemia for decades and include B2M, PTH, and FGF23. However, the exact molecule or the group of molecules that cause uraema or whether their levels relate to clinically significant negative biological events is unclear [49].

B2M, weighing 12,000 Da, is a polypeptide that forms part of the Major Histocompatibility Class I family and is usually eliminated by the kidneys, whereas kidney impairment leads to its accumulation. The accumulation of B2M has been associated with organic disease in dialysis patients (dialysis-related amyloidosis and cardiovascular disease) [50,51,52]. Studies on haemodialysis patients have demonstrated increased mortality associated with higher B2M levels, and efforts have been made to remove these molecules via improved dialysis methods (higher flux dialysers and haemodifiltration) [53,54]. However, studies have not yet shown improved long-term patient survival or reduced hospitalizations with increased B2M removal with these methods [55,56]. Similarly, in PD, serum B2M levels do not directly correlate with mortality, although serum concentrations relate to RKF: the higher the RKF, the lower the B2M levels [57]. In addition, when compared to small water-soluble molecules like urea and creatinine, the clearance of B2M in both HD and PD is significantly lower, and the traditional methods of measuring dialysis adequacy (with Kt/V and CCr) do not correlate with the clearance of middle molecules like B2M [58,59].

PTH and FGF23 (9000 and 32,000 Da, respectively) are important molecules in bone metabolism. Although recognized as a ureamic toxin, its accumulation in kidney disease is related to augmented secretion rather than decreased elimination by the kidneys. The skeletal effects of high PTH are well known in kidney disease, but studies have also demonstrated multiple extra-skeletal effects including cardiac fibrosis, decreased erythropoiesis, peripheral neuropathy, and glucose intolerance [60].

FGF23 is a phosphaturic molecule that is stimulated in CKD largely by a rising serum phosphate. High FGF23 levels, both in the intact form and as partially degraded fragments, have been found as early as Stage 2 CKD and are associated with profoundly negative biological effects and a raised mortality risk [61,62]. Efforts to eliminate high FGF23 levels using inhibitors and antibodies in humans have been largely unsuccessful [63].

## 7. Protein-Bound Uraemic Toxins

Protein-bound uraemic toxins represent about 25% of known uraemic toxins and include indoxyl sulfate (IS) and p-cresyl sulfate (PCS) [64,65,66]. Intestinal bacteria metabolize tryptophan and tyrosine and phenylalanine, respectively, to produce IS and PCS [67,68]. These molecules undergo further structural change in the liver and are normally excreted via proximal renal tubular secretion through the basolateral organic anion transporters [69,70,71,72,73]. They are both highly albumin-bound in the circulation, and it is the free concentration that is thought to induce deleterious effects [74,75]. Although total PCS and IS increase in kidney failure due to reduced renal secretion, there is reduced protein binding due to alterations in post-translational modifications of plasma proteins which increases the free toxin concentration [76,77]. This in turn results in increased free IS and PCS, as well as total PBT concentrations.

## 8. Negative Biological Effects of PBTs

PBTs have been shown to exert multiple negative systemic biological effects, leading to the progression of CKD and cardiovascular morbidity and mortality. Therefore, PBTs may have a stronger correlation with patient outcomes than traditional uraemic toxins [78,79].

IS and PCS has been shown to induce a variety of nephrotoxic effects through formation of reactive oxygen species (ROS) and induction of inflammation leading to progressive renal failure. This has been demonstrated in cellular, animal, and human studies [78,80]. PBTs increase leucocyte oxidative burst activity and ROS production that lead to activation of downstream pathways involving nuclear factor kappa-light-chain-enhancer of activated B cells (NF-κB), plasminogen activator inhibitor-1, and transforming growth factor beta 1 (TGF-β1) [81,82,83,84,85,86,87,88]. This leads to inflammation, tubulointerstitial fibrosis, and progression of kidney disease [64,85,89,90,91,92]. Niwa and colleagues demonstrated increased creatinine and glomerular sclerosis index by administrating IS to subtotally nephrectomised uraemic rats [93,94]. Other studies have also found that PBTs induce kidney tissue remodeling and fibrosis by activating epidermal growth factor receptor and epithelial–mesenchymal transition of tubular epithelial cells through activation of the renin–angiotensin–aldosterone system [95,96]. A study by Wu and colleagues found that IS and PCS was associated with renal progression, independent of other confounding factors like age, gender, diabetes, albumin, serum creatinine, calcium× phosphate product, parathyroid hormone, haemoglobin, or c-reactive protein levels [97]. Other studies by Niwa and Lin also demonstrated that PBTs increased the progression of CKD to end-stage renal failure needing dialysis [98,99]. The same modulations also delay recovery from acute kidney injury by preventing physiological processes involved in renal recovery like production of nitric oxide synthase and accelerated angiogenesis [100].

PCS and IS are also implicated in cardiovascular disease, predominantly due to arteriosclerosis, atherosclerosis, arrhythmogenesis, and congestive cardiac failure [101,102]. Similar to the pathophysiology of kidney disease, PBTs increase ROS (through induction of nicotinamide adenine dinucleotide phosphate oxidase and NF-κB/TGF-β pathways) and reduce glutathione and nitric oxide levels, leading to endothelial dysfunction [99,103,104,105,106,107]. This is further augmented by increased adhesion molecules between endothelial cells and leucocytes, resulting in activation of the immune system and further endothelial dysfunction [92,108,109,110,111]. For example, Meijers found that PCS was associated with markers of endothelial dysfunction (soluble P-selectin and endothelial microparticles) in 100 HD patients [112]. Furthermore, PBTs are shown to reduce endothelial proliferation and migration of endothelial progenitor cells which are vital in neovascularization and recovery in ischemic conditions [113,114]. PBTs also promote vascular calcification, leading to arteriosclerosis. This is primarily due to differentiation of vascular smooth muscle cells from a contractile to osteogenic phenotype by increasing expression of osteoblast-specific protein and activation of osteoblastic pathways, again through oxidative stress [99,115,116,117,118,119]. PBTs increase vascular smooth muscle cell proliferation, cardiac fibroblast collagen synthesis, and myocyte hypertrophy leading to alteration of electrical conduction and arrhythmogenesis [120,121,122,123,124,125]. Therefore, through various mechanisms described, PBTs lead to increased cardiovascular events, morbidity, and mortality. Meijers and colleagues carried out a prospective observational study in 499 patients with mild–moderate CKD and found that PCS was a predictor of cardiovascular events, independent of Framingham risk factors. Other CKD and HD studies have also demonstrated that PBTs were independently associated with cardiovascular mortality and were a useful predictor of cardiovascular events and all-cause mortality [126,127,128,129,130,131,132,133]. Similarly, a longitudinal study in PD patients found that PBTs were lower in those with RKF and that they were independently associated with cardiovascular events, all-cause mortality, and PD failure event after adjusting other confounding factors like age, diabetes, and hypertension [134].

PBTs can also negatively affect the immune system by reducing phagocytic functional capacity, granulocyte function, and protective leukocyte chemiluminescence production, leading to an increased rate of hospitalizations for infections [135,136]. They affect bone metabolism by reducing osteoblast cell formation and proliferation, bone mineralization, alkaline phosphatase activity, and expression of bone formation genes via induction of oxidative stress [137,138,139,140,141]. Finally, a study by Bammens and colleagues also found that PBTs were associated with uremic symptoms compared with other solutes where there was no relationship [142].

## 9. Measurement of PBTs

Multiple methods have been trialed to measure PBTs over the last decade, including tandem liquid chromatography/mass spectrometry (LC/MS) and HPLC (high-performance liquid chromatography), but the latter has been widely adopted more recently [143,144,145,146,147]. Additionally, research groups have further augmented their HPLC methods by developing rapid and simple sample preparation with good linearity, reversibility, reproducibility, and validity. For example, Silva and colleagues successfully applied their methods to analyze five different uraemic toxins (including PBTs) from healthy controls where they exhibited normal uraemic toxins levels and CKD patients that had significantly higher levels [148,149].

However, routine measurement of PBTs is limited by the availability of these machines in standard clinical practice and the laboratory staff expertise in carrying out these tests. In addition, running HPLC is expensive, from the cost of the machine purchase, requirement of reagents for sample analysis, and daily running costs.

## 10. Reducing Serum Protein Bound Uraemic Toxins

So far, we demonstrated the association between PBTs and abnormal cellular function and/or poor patient outcomes. To reduce serum levels of PBTs, there has been effort to enhance in elimination via dialysis, reduce absorption by absorptive agents, and reduce production using fiber supplementation.

## 11. Protein Bound Uraemic Toxin Removal in Haemodialysis

Both PCS and IS are poorly removed via HD, with studies showing that PBT removal is up to two-fold lower than clearance of urea or creatinine [150]. This relates to the binding of PBTs to albumin via reversible but strong non-covalent bonds [74] and HD clearance only permits removal of the free fraction. Deltombe and colleagues found that there was a reduction in the free PBT concentration and a rise in the proportion of protein-binding after 120 min of dialysis [151]. This is because the physicochemical bond of the toxin-albumin is so strong that the equilibrium reaction is too slow to restore the free toxin concentration within the time frame of a single passage through the dialyzer. Although studies have shown some improvement in removing PBTs with haemodialfiltration compared to standard HD, this is still not comparable to urea and creatinine removal [152]. Effectively, neither high flux dialysis nor increased dialysis appears to make a difference to PBT removal [150,153].

## 12. Protein Bound Uraemic Toxin Removal Comparing Haemodialysis and Peritoneal Dialysis

Although HD clearance of PBTs is lower, PD clearance appears worse. Evenepoel and colleagues compared patients on high-flux HD with PD and found that despite the renal clearances being higher in PD patients, the overall clearances (dialytic and renal) were significantly higher in the HD group [154]. These findings have been corroborated, with similar results in other studies [155,156].

Interestingly however, lower serum concentrations of PBTs were found in PD patients compared to HD despite HD patients demonstrating higher clearance. This could be explained by the intermittent nature of HD (three times/week for 4–5 h) compared with PD (potentially longer therapy and more often), allowing for slow equilibrium of the free PBTs to move across the peritoneal membrane. Additional factors include altered intestinal generation and/or metabolism of PBTs or protein loss during dialysis in PD patients [157,158] and impaired nutrition and/or delayed colonic transit in HD patients [159,160].

## 13. Removal of PBTs by Peritoneal Dialysis

PBT clearances in PD are 10-fold lower than that of urea and/or creatinine [142,158]. This is because PBT clearance primarily occurs through RKF, where elimination of PBTs depends largely on kidney specific mechanisms like tubular secretion which cannot be achieved with dialysis [161]. This is demonstrated in multiple studies over the last two decades. Bammens in 2003 performed a cross-sectional observational study on 30 PD patients with RKF where they found that renal clearance of PCS contributed 61.9 ± 4.6% of the total clearance [142]. A longitudinal study of 24 PD patients by the same research group found that RKF reduced over their 7-month study period, with concurrent increases in peritoneal clearance of water-soluble uraemic toxins due to increased PD prescription by physicians (to compensate for reduced RKF) [161]. However, no increase in peritoneal clearance of PCS was demonstrated with peritoneal clearance contributing only 1/10th of total clearance. Similar findings have been reported in later studies, where the renal clearances of IS and PCS were up to 8.5-fold higher than their peritoneal clearances [155,162].

Although earlier observational studies showed improved PBT clearances with continuous ambulatory PD compared with automated PD [154,163], later studies have demonstrated no significant differences [164,165]. Similarly, no differences in PBT removal were found between different peritoneal membrane transport groups [162].

Despite PBTs being poorly cleared by PD, it is unclear what happens to the serum PBT levels once RKF declines in PD patients. While earlier studies did not demonstrate a parallel increase in serum PBT levels with declining RKF [157,163], later studies have shown up to a 20% increase in serum levels in those patients without RKF [166].

## 14. Reducing PBT Absorption

Given that PBTs are poorly removed by dialysis, there has been an effort looking into substances that reduce PBT absorption to reduce serum PBT concentrations. AST-120 is an oral charcoal absorbent that initially was thought to reduce serum PBT levels [167,168] resulting in reduced progression of glomerular sclerosis [169,170,171], improved cardiac hypertrophy and fibrosis [172,173,174] and mortality [175], but these findings were not consistently reproduced in subsequent studies [176,177]. Other substances, such as Shen-Shuai-Ning [178] and ibuprofen—a competitive binder of PBTs to albumin [179,180]—have also shown a reduction in PBTs. However, studies in this area are limited, and given the lack of reproducibility and/or the risk of systemic side effects, these agents have not been adopted widely in clinical practice.

## 15. Reducing PBT Generation

Another possible way of reducing PBTs in serum is to reduce their generation. Diet and dietary fiber have been shown to reduce PBTs in patients with renal impairment. This could be due to a multitude of reasons, including increased intestinal motility [181], thus decreasing toxin absorption and increasing fecal excretion, improving the integrity of tight junctions in the colonic epithelium (to reduce PBT permeability), and facilitating the growth of a more favorable microbiome [182,183]. Despite such potential benefits, studies have found that the average fiber intake in dialysis patients is 20–30% lower than for control patients [184], with the daily recommended amount in patients with CKD being 25–35 g/day [185,186].

Dietary fiber can be grouped as pre-biotic (non-fermented ingredient with fiber-like properties), pro-biotic (containing live microorganisms which, administered in adequate amounts, can balance the intestinal microbiota profile), or symbiotic (use of a combination of pro- and pre-biotics) [187,188]. Studies in CKD patients reveal mixed results while HD patients do appear to reduce PBT levels with fiber supplementation. Salmean and colleagues carried out a study on CKD patients (eGFR < 50 mL/min/1.73 m^2^) supplementing two types of fiber over 12 weeks [189]. He found that fiber supplementation decreased PCS levels by 20%. Similarly, most CKD studies have also demonstrated a reduction in PBT levels up to 40% as quickly as after 2 weeks of treatment with fiber, an effect that persisted for up to 4 weeks [190,191,192,193,194,195]. However, other studies on CKD patients yielded either non-significant [196,197,198] or negative results [199,200].

A majority of studies on HD patients suggest that PBT levels are reduced with fiber supplementation. Sirich and colleagues randomized HD patients to receive fiber or controlled starch for 6 weeks and found a reduction in free IS and PCS levels by 29% and 28%, respectively [201]. Similarly, other HD studies on the use of fiber in the form of pre-biotics, pro-biotics, and synbiotics demonstrated a reduction in PBTs from between 10 and 54% as quickly as after 4 weeks of treatment [202,203,204,205,206,207,208,209,210,211,212]. However, no studies have determined whether this concurrently improved patient symptoms and/or mortality. The single study in PD patients was a randomized crossover trial where fiber supplementation failed to change serum PBT concentrations after 12 weeks of treatment [213]; however, no subsequent studies have been performed. Future studies need to determine whether fiber supplementation in PD patients can reduce PBTs and whether this influences patient outcomes.

## 16. Conclusions and Directions for the Future

In this review, we outlined why the traditional measurements of dialysis adequacy using urea and/or creatinine have been abandoned in the PD sphere. This has led to a change in the ISPD guidelines to focus on patients’ well-being and symptoms as a surrogate marker of PD adequacy. Evidence in the last two decades has demonstrated that PBTs might have a stronger correlation with patient outcomes compared with traditional uraemic toxins, contributing to abnormal cellular function and patient mortality. However, widespread use of PBTs in measuring dialysis adequacy has been limited by its methods of analysis using HPLC. PBTs have been consistently shown to be poorly cleared via PD with research now focusing on PBT absorption and reducing generation. Future studies need to determine whether measurement of PBTs can replace current use of traditional measures and whether reduction in PBTs improves patient outcomes including patients’ symptoms and well-being.

## Figures and Tables

**Table 1:** Methods of measuring total Kt/V and creatinine clearance (CCr).

Kt/V	Creatinine Clearance (CCr)
Peritoneal Kt/V Daily Kt = (D_urea_/P_urea_) × 24 h dialysate volume V = Total body water estimated by Watsons Formula Weekly peritoneal Kt/V = Daily peritoneal Kt/V × 7	Daily peritoneal creatinine clearance = 24 h dialysis creatinine/Serum creatinine Weekly peritoneal clearance = Daily peritoneal clearance × 7
Renal Kt/V Daily Kt = (U_urea_/P_urea_) × 24 h urine volume V = Total body water estimated by Watsons formula Weekly renal Kt/V = Daily renal Kt/V × 7	Daily renal creatinine clearance = 0.5 × (24 h urine creatinine/serum creatinine) + (24 h urine urea/serum urea) Weekly renal clearance = Daily renal clearance × 7
Total weekly Kt/V = Weekly peritoneal Kt/V + Weekly renal Kt/V	Total weekly creatinine clearance = Weekly peritoneal clearance + Weekly renal clearance calculated for body surface area 1.73 m^2^ using DuBois method

## Abbreviations

The following abbreviations are used in this manuscript:

PD	Peritoneal dialysis
PBTs	Protein-bound uraemic toxins
HD	Hemodialysis
ISPD	International Society for Peritoneal Dialysis
CKD	Chronic kidney disease
CCr	Creatinine clearance
RKF	Residual kidney function
IS	Indoxyl sulfate
PCS	p-cresyl sulfate
ROS	Reactive oxygen species
NF-κB	Nuclear factor kappa-light-chain-enhancer of activated B
TGF-β1	Transforming growth factor beta 1
LC/MS	Liquid chromatography/Mass spectrometry
HPLC	High-performance liquid chromatography
